# Survival analysis between different treatment strategies of mixed adenoneuroendocrine carcinoma (MANEC): a population-based study

**DOI:** 10.1530/EC-24-0350

**Published:** 2025-02-18

**Authors:** Wenkai Li, Jing Wang, Jie Lin, Lei Sun, Manzhao Ouyang, Jie Ding, Cuifang Han, Binbin Li, Guo-Liang Huang

**Affiliations:** ^1^ Guangdong Provincial Key Laboratory of Medical Immunology and Molecular Diagnostics, The First Dongguan Affiliated Hospital, Guangdong Medical University, Dongguan, China; ^2^ Cancer Center, The First Dongguan Affiliated Hospital, Guangdong Medical University, Dongguan, China; ^3^ Department of Gastrointestinal Surgery, Shunde Hospital, Southern Medical University (The First People’s Hospital of Shunde Foshan), Foshan, Guangdong Province, China

**Keywords:** mixed adenoneuroendocrine carcinoma, MANEC, survival, prognosis, treatment

## Abstract

**Background:**

Mixed adenoneuroendocrine carcinoma (MANEC) is rare and heterogeneous. Currently, there is no concise treatment standard for MANEC. This study aimed to explore the prognostic impact of different treatment methods in MANEC.

**Methods:**

The surveillance, epidemiology and end results database was utilized to evaluate the outcomes of MANEC using propensity score matching (PSM) , stratified and site-specific survival analysis.

**Results:**

A total of 935 patients diagnosed with MANEC were enrolled in this study. Across most analyses, surgery performance (compared with no surgery) was associated with improved survival outcomes for MANEC patients, except for the site-specific analysis of small intestine, in which no significant association between surgery and patients’ survival was found. Radiotherapy exhibited no significant association with patients’ survival in most cases, and associated with poor prognosis of MANEC in certain specific analyses. Inconsistent effects of chemotherapy on MANEC were observed. Positive impact of chemotherapy on MANEC was indicated in PSM for chemotherapy or radiotherapy. Contradictory effects of chemotherapy were suggested based on the stage of MANEC, with unfavorable outcomes in early-stage and favorable outcomes in advanced-stage. The impact of chemotherapy on survival was influenced by the administration of surgery or radiotherapy. Site-specific analyses indicated that chemotherapy was beneficial to MANEC of the hepatic–biliary–pancreatic system.

**Conclusions:**

The results of this study demonstrate the variable impact of different treatment strategies for MANEC. Surgery was generally associated with improved survival outcomes, while radiotherapy did not show a beneficial effect on MANEC. The effects of chemotherapy on MANEC were inconsistent in specific analyses.

## Introduction

Neuroendocrine neoplasms (NENs) are a group of rare malignant neoplasms, which encompass well-differentiated neuroendocrine tumors, poorly differentiated neuroendocrine carcinomas (NECs) and mixed neuroendocrine–nonNENs (MiNENs) (1, 2). The term ‘mixed neuroendocrine–nonneuroendocrine neoplasm (MiNEN)’ was proposed in 2016 to define and to unify the concept of a heterogeneous group of neoplasms characterized by the presence of both neuroendocrine and nonneuroendocrine components (3, 4). This definition of MiNEN was subsequently included in the 2019 World Health Organization (WHO) classification, maintaining both the neuroendocrine and nonneuroendocrine components were required to account for more than 30% of the total tumor amount (4, 5).

Mixed adenoneuroendocrine carcinoma (MANEC), as a major subtype of MiNEN, consists of both adenocarcinoma (ACA) and NEC components. In the 2000 WHO classification, it was initially categorized as mixed exocrine–endocrine tumors and subsequently replaced by MANEC in 2010 (4). MANEC is a neoplasm that exhibits notable histological heterogeneity (6). Inconsistent conclusions have been reported regarding the prognosis of MANEC. Several studies have indicated that the prognosis was intermediate between those of the two ‘pure’ components constituting it. However, other investigations have discovered that both MANEC and pure NEC have worse prognoses compared to pure ACA (7, 8, 9, 10).

In addition to tumor component, many factors, such as tumor grade and stage, significantly influence the prognosis and management of MANEC (4). It is important to note that there is currently no concise guideline or treatment standard for MANEC due to the rarity, heterogeneity and limited literature (2). Different regimens are determined by either the predominant histological component or the more aggressive one (7, 9, 11). Nevertheless, several studies indicated that MANEC can be managed in the same way as conventional ACA, considering that its developmental connection with ACA is stronger than that with NEC counterparts (12, 13). Therefore, it is important to explore the prognostic impact of different treatment methods in MANEC. This study utilized the surveillance, epidemiology and end results (SEER) database to assess the outcomes of MANEC patients through propensity score matching (PSM) , stratified and site-specific survival analysis.

## Patients and methods

### Data source and patient selection

**Figure 1:**
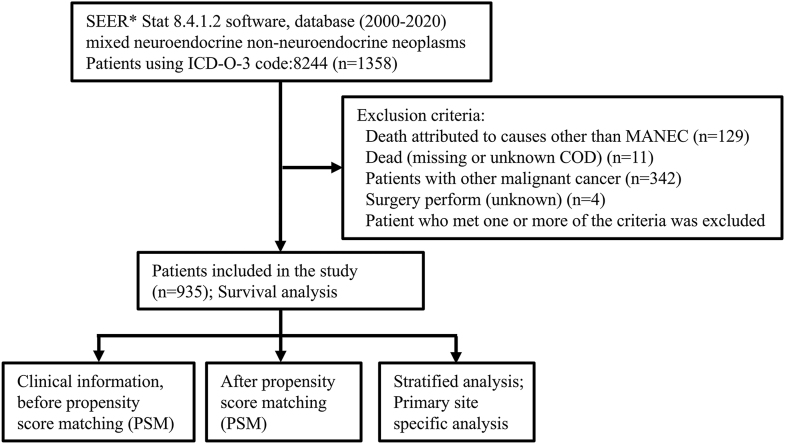
Flowchart of database mining and patient selection.

Data for patients diagnosed with MANEC between 2000 and 2020 were obtained from the SEER research data (17 registries, SEER database (2000–2020)). The treatment strategies, clinicopathological information and survival data of the MANEC patients were extracted using the SEER*Stat 8.4.1.2 software (https://seer.cancer.gov/seerstat/download/). The following mining strategies were employed for the SEER database: ‘cases from the research database’, ‘year of diagnosis between 2000 and 2020’, ‘ICD-O-3 code = 8244’ and ‘diagnostic confirmation = microscopic examination’. The study enrolled participants who meet the following selection criteria: i) patients who received a pathological diagnosis of MANEC between January 1, 2000 and December 31, 2020 and ii) patients who had active follow-up and did not present any other type of cancer. The exclusion criteria included: i) patients who died due to causes unrelated to MANEC; ii) patients with missing or unknown cause of death information; and iii) patients diagnosed with other types of malignant tumors. A detailed and comprehensive overview of the data extraction process is provided in Fig. 1.

The following clinicopathological data were extracted from the SEER database: patient ID, age, sex, race, year of diagnosis, site according to ICD-O-3/WHO 2008, vital status, survival months, SEER cause-specific death classification, differentiation grade, summary stage, AJCC stage, surgery, the reason for no cancer-directed surgery, chemotherapy, radiotherapy, COD to site and the total number of *in situ*/malignant tumors for patient.

### Statistical analysis

Continuous variables were provided as the mean ± standard deviation or median (range). Differences between groups were assessed with *T* test or Mann–Whitney *U* test. Categorical data were analyzed with the Chi-square test. Survival curves were generated using the Kaplan–Meier method and differences between groups were evaluated using log-rank test. The primary endpoint for the survival analysis was cancer-specific mortality, which referred to deaths attributed to MANEC as indicated by the recorded cause of death from the SEER database. All statistical analyses mentioned above were conducted using the R4.1.2 software with ‘MatchIt’, ‘tableone’, ‘survival’, ‘survminer’ and other language packages. For PSM analysis, a caliper width of 0.02 was set and the 1:1 nearest neighbor matching method was employed. All tests were two-sided and *P* value <0.05 was considered statistically significant.

## Results

### Patient characteristics

After patient selection, a total of 935 patients diagnosed with MANEC in the SEER database from 2000 to 2020 were included in this study. The baseline characteristics of all patients are summarized in Supplementary Table S1 (see section on Supplementary materials given at the end of the article). Of the overall population, 481 (51.4%) were male and 454 (48.6%) were female, with mostly Caucasians (*n* = 758, 81.4%), and a median age at diagnosis of 60 years. A large proportion of MANEC were found in the appendix (*n* = 482, 51.6%) and the colorectum (*n* = 223, 23.9%). After excluding 545 cases with unknown data of the 6th AJCC staging, 163 (41.8%) were identified in the stage I and II, while 227 (58.2%) were in the stage III and IV. After excluding 452 cases with no information of differentiation grade, 149 (30.8%) were classified as grade I and II, while 334 (69.2%) were classified as grade III and IV. Regarding the treatment strategies of MANEC, surgery was performed in 815 (87.2%) patients, chemotherapy was administered to 495 (52.9%) patients and radiation therapy was provided to only 61 (6.5%) patients.

### Survival analysis of overall participants

**Figure 2:**
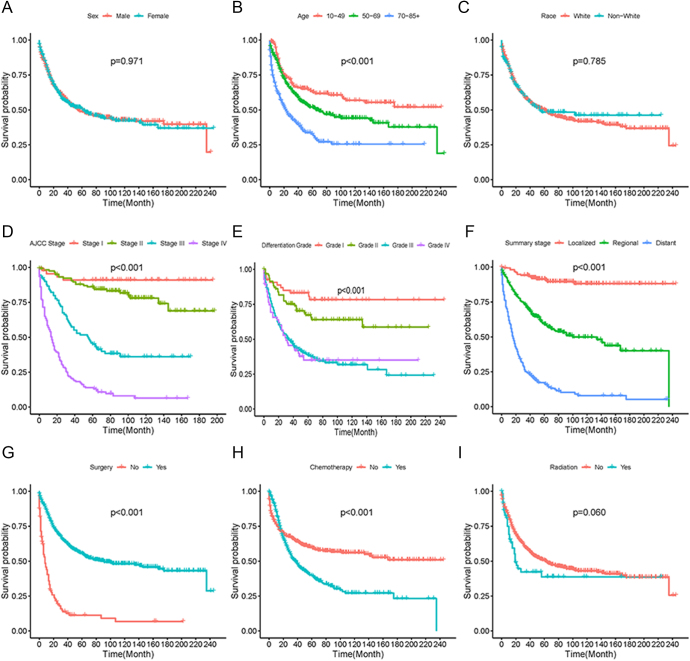
Survival analysis of overall participants. Survival analysis based on sex (A), age (B), race (C), AJCC stage (D), differentiation (E), summary stage (F), surgery (G), chemotherapy (H) and radiation (I).

Survival analyses were conducted with overall participants to investigate the differences in survival rates based on various clinicopathological characteristics (Fig. 2). The results revealed no significant differences in survival rates based on gender, race or the treatment of radiation therapy or not. A younger age at diagnosis was associated with a higher survival rate compared to older individuals. Furthermore, survival analyses of AJCC stage, differentiation grade and summary stage suggested that an early stage or low grade was associated with a more favorable prognosis in the overall population. Participants who underwent surgery demonstrated better survival outcomes, whereas those receiving chemotherapy showed a poorer prognosis.

### Survival analysis in PSM cohort

**Figure 3:**
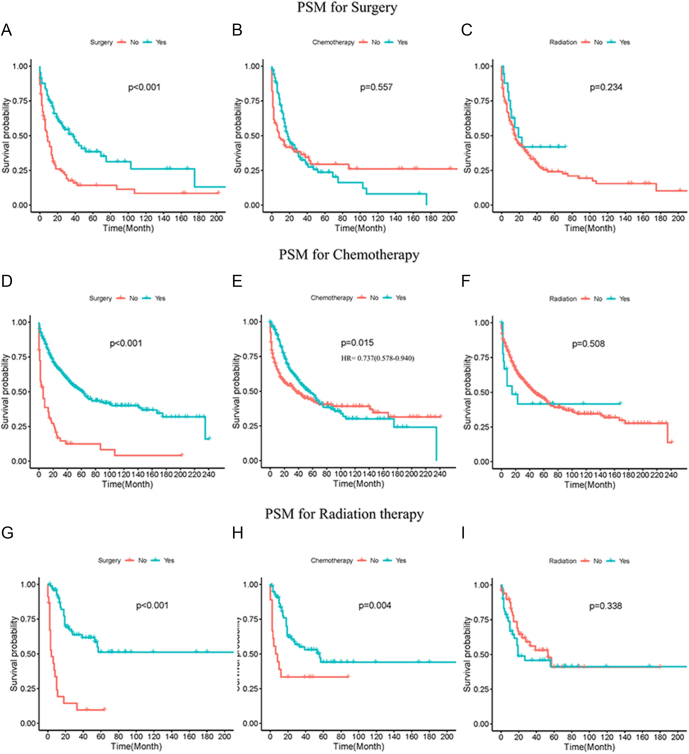
Survival analysis of propensity score matched (PSM) cohort. Survival analysis based on surgery (A), chemotherapy (B) and radiation (C) using PSM cohort for ‘surgery’. Survival analysis based on surgery (D), chemotherapy (E) and radiation (F) using PSM cohort for ‘chemotherapy’. Survival analysis based on surgery (G), chemotherapy (H) and radiation (I) using PSM cohort for ‘radiation’.

PSM is able to reduce selection bias in non-randomized studies and to balance covariates among different treatment groups (14). In this study, the various therapy groups, including ‘surgery’ versus ‘no surgery’, ‘chemotherapy’ versus ‘no chemotherapy’ and ‘radiation therapy’ versus ‘no radiation therapy’, were matched using PSM methods (Supplementary Figs S1, S2, S3). A total of 178 participants were successfully matched after PSM for ‘surgery’ (Table 1). Survival analyses using this cohort indicated that surgery performance was beneficial to patients’ survival, while chemotherapy or radiation therapy administration provided no benefits to patients’ survival (Fig. 3A, B, C). After performing PSM for ‘chemotherapy’, a total of 522 participants were matched and enrolled in the cohort (Table 2). The results of survival analyses showed that surgery performance had a positive effect on patients’ survival, while radiation therapy administration did not provide any survival benefits (Fig. 3D, E, F). Although survival analysis between chemotherapy and ‘no chemotherapy’ yielded a *P* value less than 0.05, the survival curve suggested that chemotherapy might be beneficial for survival, especially in short-term survival times (HR = 0.737(0.578–0.940), *P* = 0.014, Fig. 3D, E, F). A cohort with 98 patients was matched after PSM for ‘radiation therapy’ (Table 3). The survival analyses data demonstrated that surgery or chemotherapy administration had a positive impact on patients’ survival, while radiation therapy had no benefits for patients’ survival (Fig. 3G, H, I).

**Table 1:** Clinicopathological characteristics of patients before and after PSM for surgery (cases (%)).

Characteristics	Before PSM (935)			After PSM (178)		
	No surgery	Surgery	*P* value	No surgery	Surgery	*P* value
Sex			0.129			1.000
Male	70(58.3)	411(50.4)		50(56.2)	50(56.2)	
Female	50(41.7)	404(49.6)		39(43.8)	39(43.8)	
Age (years)			0.033			1.000
<60	46(38.3)	401(49.2)		38(42.7)	38(42.7)	
≥60	74(61.7)	414(50.8)		51(57.3)	51(57.3)	
Race			1.000			0.137
White	97(81.5)	661(81.4)		71(80.7)	79(89.8)	
Non-White	22(18.5)	151(18.6)		17(19.3)	9(10.2)	
AJCC staging (6th)			<0.001			0.806
I + II	6(14.0)	157(45.2)		6(21.4)	5(15.6)	
III + IV	37(86.0)	190(54.8)		22(78.6)	27(84.4)	
Differentiation grade			0.035			0.694
Grade I + II	5(13.9)	144(32.2)		4(13.8)	3(8.8)	
Grade III + IV	31(86.1)	303(67.8)		25(86.2)	31(91.2)	
Summary stage			<0.001			0.861
Localized	7(9.7)	149(25.6)		7(13.7)	9(16.1)	
Regional	7(9.7)	251(43.0)		7(13.7)	6(10.7)	
Distant	58(80.6)	183(31.4)		37(72.5)	41(73.2)	
Chemotherapy			0.172			0.880
No	49(40.8)	391(48.0)		38(42.7)	40(44.9)	
Yes	71(59.2)	424(52.0)		51(57.3)	49(55.1)	
Radiation therapy			<0.001			1.000
No	96(80.0)	776(95.5)		80(89.9)	81(91.0)	
Yes	24(20.0)	37(4.5)		9(10.1)	8(9.0)	

**Table 2:** Clinicopathological characteristics of patients before and after PSM for chemotherapy (cases (%)).

Characteristics	Before PSM (935)			After PSM (522)		
	No chemotherapy	Chemotherapy	*P* value	No chemotherapy	Chemotherapy	*P* value
Sex			0.052			0.539
Male	211(48.0)	270(54.5)		135(51.7)	143(54.8)	
Female	229(52.0)	225(45.5)		126(48.3)	118(45.2)	
Age (years)			0.006			0.597
<60	189(43.0)	258(52.1)		113(43.3)	120(46.0)	
≥60	251(57.0)	237(47.9)		148(56.7)	141(54.0)	
Race			0.488			1.000
White	352(80.4)	406(82.4)		214(82.6)	215(82.7)	
Non-White	86(19.6)	87(17.6)		45(17.4)	45(17.3)	
AJCC staging (6th)			<0.001			1.000
I + II	127(63.8)	36(18.8)		29(31.2)	32(32.0)	
III + IV	72(36.2)	155(81.2)		64(68.8)	68(68.0)	
Differentiation grade			<0.001			0.603
Grade I + II	94(42.5)	55(21.0)		30(24.2)	37(27.8)	
Grade III + IV	127(57.5)	207(79.0)		94(75.8)	96(72.2)	
Summary stage			<0.001			0.575
Localized	122(37.3)	34(10.4)		22(13.3)	29(16.6)	
Regional	126(38.5)	132(40.2)		72(43.6)	79(45.1)	
Distant	79(24.2)	162(49.4)		71(43.0)	67(38.3)	
Surgery			0.172			0.301
No	49(11.1)	71(14.3)		39(14.9)	30(11.5)	
Yes	391(88.9)	424(85.7)		222(85.1)	231(88.5)	
Radiation therapy			<0.001			
No	431(98.0)	441(89.4)		252(96.6)	251(96.2)	1.000
Yes	9(2.0)	52(10.6)		9(3.4)	10(3.8)	

**Table 3:** Clinicopathological characteristics of patients before and after PSM for radiation therapy (cases (%)).

Characteristics	Before PSM (933)			After PSM (98)		
	Radiation therapy		*P* value	Radiation therapy		*P* value
	No	Yes		No	Yes	
Sex			0.062			0.393
Male	442(50.7)	39(63.9)		35(71.4)	30(61.2)	
Female	430(49.3)	22(36.1)		14(28.6)	19(38.8)	
Age (years)			0.875			1.000
<60	417(47.8)	28(45.9)		23(46.9)	22(44.9)	
≥60	455(52.2)	33(54.1)		26(53.1)	27(55.1)	
Race			0.080			0.814
White	712(82.0)	44(72.1)		38(77.6)	36(73.5)	
Non-White	156(18.0)	17(27.9)		11(22.4)	13(26.5)	
AJCC staging (6th)			0.241			1.000
I + II	158(42.7)	5(26.3)		3(25.0)	5(27.8)	
III + IV	212(57.3)	14(73.7)		9(75.0)	13(72.2)	
Differentiation grade			0.973			0.440
Grade I + II	140(31.0)	9(29.0)		11(40.7)	7(26.9)	
Grade III + IV	311(69.0)	22(71.0)		16(59.3)	19(73.1)	
Summary stage			0.752			0.281
Localized	149(24.1)	7(18.9)		7(24.1)	6(18.2)	
Regional	243(39.4)	15(40.5)		16(55.2)	14(42.4)	
Distant	225(36.5)	15(40.5)		6(20.7)	13(39.4)	
Chemotherapy			<0.001			1.000
No	431(49.4)	9(14.8)		9(18.4)	9(18.4)	
Yes	441(50.6)	52(85.2)		40(81.6)	40(81.6)	
Surgery			<0.001			
No	96(11.0)	24(39.3)		8(16.3)	14(28.6)	0.226
Yes	776(89.0)	37(60.7)		41(83.7)	35(71.4)	

### Stratified survival analysis

**Figure 4:**
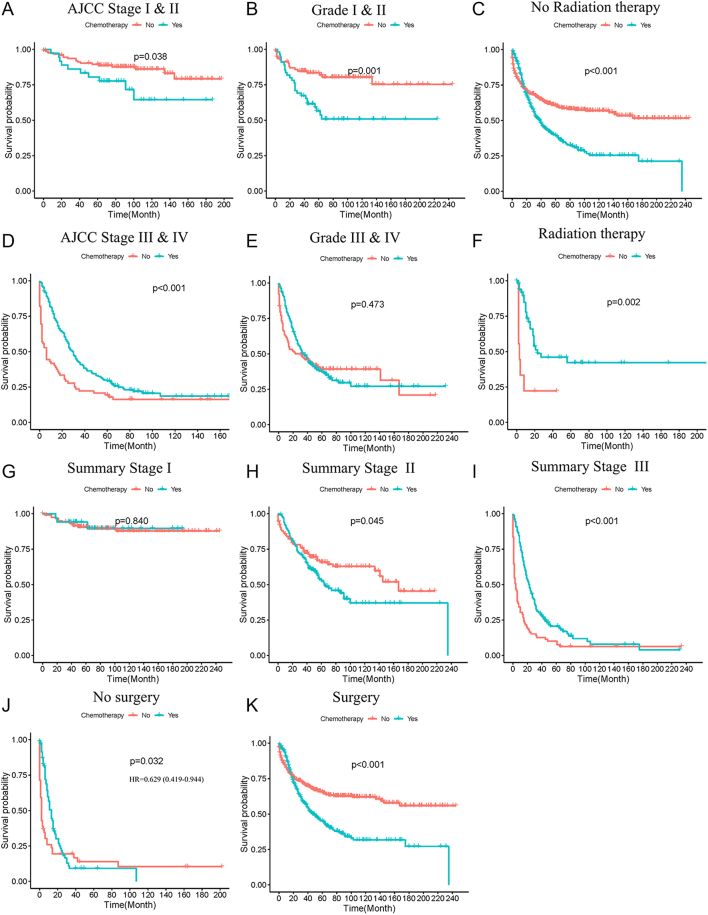
Stratified survival analysis of chemotherapy in population with different clinicopathological characteristics. Stratified survival analysis of chemotherapy in patients of AJCC stage I and II (A), AJCC stage III and IV (D), grade I and II (B), grade III and IV (E), no radiation therapy (C), radiation therapy (F), summary stage I (G), summary stage II (H), summary stage III (I), no surgery (J) and surgery (K).

Meaningful findings were suggested in the stratified survival analysis of patients with different clinicopathological characteristics. Surgery performance was beneficial to patients’ survival in almost all stratified analyses (data not shown). The administration of chemotherapy was associated with unfavorable survival in patients with AJCC stage I and II or with summary stage II, but it was associated with favorable survival in patients with AJCC stage III and IV or with summary stage III (Fig. 4A, D, G, H, I). Chemotherapy was associated with worse prognosis in patients with differentiation grade I and II, while it was not associated with prognosis in patients with differentiation grade III and IV (Fig. 4B and E). The application of chemotherapy had a negative effect on the survival of patients without radiation therapy, but had a positive effect on patients with radiation therapy (Fig. 4C and F). Chemotherapy showed a positive impact on the survival of patients without surgery, but had a negative impact on patients with surgery (Fig. 4J and K).

**Figure 5:**
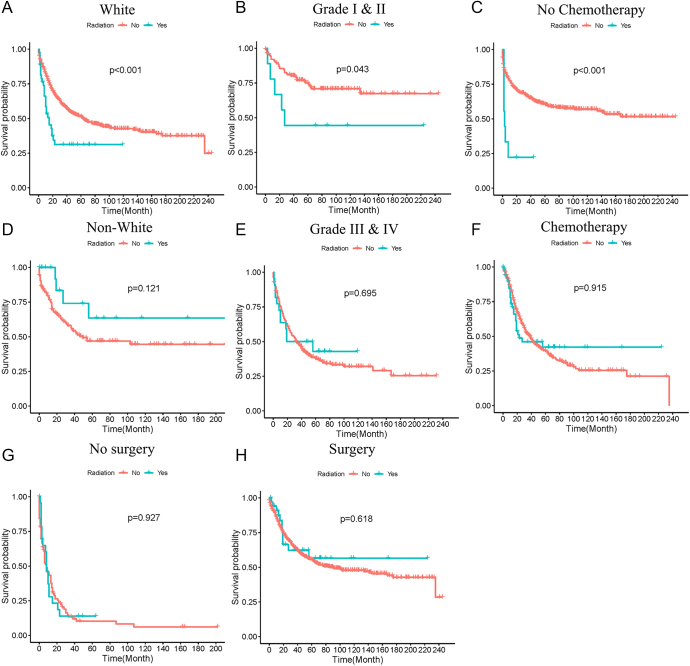
Stratified survival analysis of radiation therapy in population with different clinicopathological characteristics. Stratified survival analysis of radiation therapy in patients of White (A), non-White (D), grade I and II (B), grade III and IV (E), no chemotherapy (C), chemotherapy (F), no surgery (G) and surgery (H).

Radiation therapy was associated with worse survival in patients of White ethnicity, while it tended to be associated with better survival in patients of non-White ethnicity (*P* value = 0.121, Fig. 5A and D). The application of radiation therapy was associated with unfavorable prognosis in patients with differentiation grade I and II, while it was not associated with prognosis in patients with differentiation grade III and IV (Fig. 5B and E). The administration of radiation therapy had a negative effect on the survival of patients without chemotherapy, whereas it was not associated with survival of patients with chemotherapy (Fig. 5C and F). Radiation therapy showed no significant association with the survival of MANEC regardless of whether surgery was performed or not (Fig. 5G and H).

### Site-specific survival analysis

**Figure 6:**
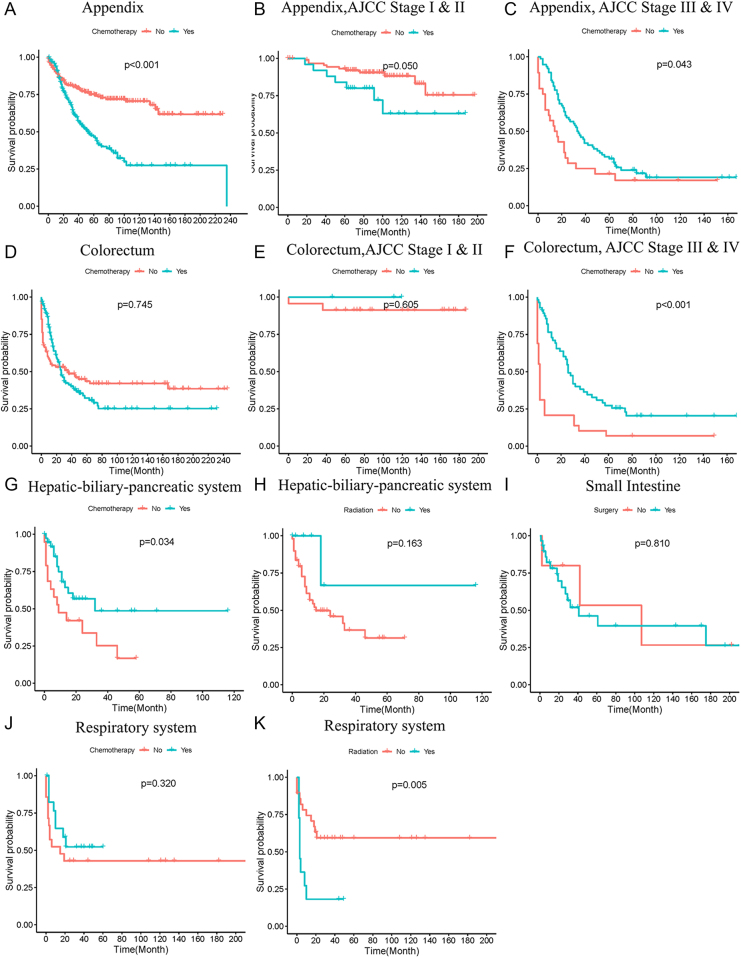
Site-specific survival analysis. Survival analysis of chemotherapy in MANEC of appendix (A), appendix with AJCC stage I and II (B), appendix with AJCC stage III and IV (C), colorectum (D), colorectum with AJCC stage I and II (E), colorectum with AJCC stage III and IV (F), hepatic–biliary–pancreatic system (G) and respiratory system (J). Survival analysis of radiation therapy in MiNENs of hepatic–biliary–pancreatic system (H) and respiratory system (K). Survival analysis of surgery in small intestine.

Site-specific analyses were conducted to examine the response of MANEC occurring in various organs to different treatment methods. The results indicated that surgery performance was beneficial to survival of patient with MANEC in most organs (data not shown), except for the small intestine, whereas there were no differences in survival rates between the surgery group and the ‘no surgery’ group (Fig. 5I). In appendix MANEC, chemotherapy was associated with worse prognosis. Further analyses revealed that chemotherapy of appendix MANEC tended to be associated with unfavorable survival in patients with AJCC stage I and II, but associated with favorable survival in patients with AJCC stage III and IV (Fig. 6A, B, C). Chemotherapy of colorectal MANEC did not affect patients’ survival in overall colorectal MANEC and in patients with AJCC Stage I and II. However, the application of chemotherapy was associated with favorable survival in colorectal MANEC with AJCC stage III and IV (Fig. 6D, E, F). For MANEC occurring in the hepatic–biliary–pancreatic system, the use of chemotherapy was associated with favorable survival (Fig. 6G and H). In MANEC of respiratory system, radiation therapy had a negative impact on patients’ survival (Fig. 6J and K).

## Discussion

Although MANEC occur in a variety of organs including appendix, colorectum, stomach, intestine, pancreas, biliary tract, cervix and lung, comprehensive study and current understanding of MANEC is limited due to the novelty of this concept and the rarity of the tumor (4). The clinical characteristic, prognosis and treatment guidelines of MANEC have yet to be fully clarified. Because of the low incidence rate of MANEC, it is challenged to conduct prospective randomized-controlled trials to optimize its clinical management. As one of the largest cancer registration databases encompassing approximately 30% of the US population, the SEER database provides a valuable resource for retrospective studies of MANEC at the population-level (15).

With the utility of SEER database, two population-based studies were conducted and focused on the analysis of MANEC in specific organs. Zhao and coworkers identified 513 patients with MANEC of the appendix and colorectum from SEER database. The results suggested that tumor location had a significant prognostic value for MANEC. More aggressive biological characteristics and a poorer prognosis were indicated in MANEC of the colorectum compared to that of the appendix (16). In the study conducted by Wang and coworkers, a total of 581 patients diagnosed with gastrointestinal MANEC were enrolled. The data revealed that the survival rate of patients with appendiceal MANEC was better than those with cecal MANEC (17). Brathwaite and coworkers conducted a comparison of the survival rates among MANEC, carcinoid tumor, signet ring cell carcinoma and goblet cell carcinoid tumors of the appendix. The outcomes indicated that MANEC represents a more aggressive clinical type in comparison to both carcinoid tumor and goblet cell carcinoid tumors of the appendix (18). Moreover, several retrospective studies were performed to reveal the epidemiological trend and the clinicopathological characteristics of MANEC. The finding from a multicenter cohort study indicated that patients with gastric MANEC exhibited poorer prognoses and a higher propensity for distant recurrence in contrast to those with gastric ACA (19). In the colorectum, the median survival of MANEC patients was more favorable compared to that of patients with primary signet-ring cell cancer in stage III-IV (20).

In our population-based study, a total of 935 cases were analyzed using the SEER database, making it the largest population-based study on the prognosis of different treatment methods of MANEC to the best of our knowledge. Our data suggested that the appendix was the most common site for MANEC, which was consistent with the studies focused on lower gastrointestinal tract (16, 21). Advanced grade or stage account for more than half of the cases with information, which was consistent with the previous study conducted on gastrointestinal tract (17). Survival analyses of overall participants revealed that a more favorable prognosis was associated with younger age at diagnosis, early AJCC stage, lower grade of differentiation and the administration of surgery. Nevertheless, the use of chemotherapy for MANEC was associated with a worse prognosis in the overall population, while the utilization of radiotherapy did not significantly impact patient outcomes.

To further evaluate the survival outcomes of different treatment strategies for MANEC, PSM, stratified and site-specific analyses were conducted. Surgical resection was the preferred treatment strategy in the management of MANEC (4, 22). Across most analyses, surgery was associated with improved survival outcomes for MANEC patients. However, in the site-specific analysis focusing on the small intestine, no significant association between surgery and patients’ survival was found, possibly due to the limited sample size in this subgroup. Radical surgery combined with multimodal therapy, including chemotherapy and radiotherapy, was suggested to improve long-term survival of MiNEN according to a multicentral study (23). The study of Zhu and coworkers suggested that postoperative chemotherapy and radiotherapy conferred benefits upon patients of colorectal MANEC (20). However, our data revealed no significant association between radiotherapy and patients’ survival in most cases. Moreover, radiotherapy was associated with poor prognosis of MANEC in certain specific analyses. These differences might be caused by the different pathological characteristics of the population.

Chemotherapy plays a crucial role in inhibiting tumor growth by disrupting DNA and RNA production, damaging DNA structure and affecting the function of proteins within tumor cells. However, chemotherapy also affects rapidly dividing normal cells, including immune cells, potentially leading to immune suppression, infections and tumor recurrence (24, 25). Consequently, chemotherapy can cause severe side effects, such as vincristine-induced peripheral neuropathy and doxorubicin-induced cardiotoxicity (26, 27). Currently, research specifically investigating the effects of chemotherapy on MANEC remains limited. A nationwide cohort study was conducted with 49 NEC and MANEC patients of esophageal or gastric cancer. The data implied that survival curves exhibited similarity regardless of whether neoadjuvant therapy was applied or not (28). The study of Turri-Zanoni and coworkers suggested that neoadjuvant chemotherapy was correlated with better survival for NEC (29). Our data showed inconsistent effects of chemotherapy on MANEC. Propensity score matched cohorts for chemotherapy or radiotherapy showed a positive impact of chemotherapy on MANEC prognosis. The effects of chemotherapy showed contradictory effects based on the stage of MANEC, with unfavorable outcomes in early-stage but favorable outcomes in advanced-stage. The impact of chemotherapy on survival was influenced by the administration of surgery or radiotherapy. Site-specific analyses indicated that chemotherapy was beneficial to MANEC of the hepatic–biliary–pancreatic system.

Several limitations of this study should be noticed. First, it was a retrospective study, which might have a bias in patient selection. Although the PSM method was used, many confounding factors might not be considered, which might lead to deviation in the data. Second, the public SEER database did not include detailed information about chemotherapy and radiotherapy, such as chemotherapy regimen, adjuvant or neoadjuvant treatment and detailed radiotherapy regimen. Other clinicopathological variables were limited in the study. Information such as immunohistochemical markers, the ki-67 index and metastatic tumor burden was not included. Third, there were relatively small sample sizes in some specific survival analyses. Despite the limitations of this study, it is the largest retrospective study to analyze the prognosis of different treatment strategies of MANEC. Our findings provide valuable insights into the treatment strategies and prognosis of MANEC based on different clinicopathological characteristics and organ sites. The administration of chemotherapy or radiotherapy for MANEC should be approached with careful consideration. However, further research is needed to validate and explore these associations in larger and more diverse patient cohorts.

## Supplementary materials



## Declaration of interest

The authors declare that there is no conflict of interest that could be perceived as prejudicing the impartiality of the work reported.

## Funding

This research was funded by the Guangdong Basic and Applied Basic Research Foundation (no. 2023A1515140159 to GLH; no. 2023A1515010448 to BL), the Dongguan Science and Technology of Social Development Program (no. 20231800940582 to GLH; no. 20231800936042 to BL), the Discipline Construction Project of Guangdong Medical University (no. 4SG24029G to GLH) and the Talent Development Foundation of the First Dongguan Affiliated Hospital of Guangdong Medical University (no. PF100-2-03 to GLH).

## Author contribution statement

Study concepts and study design were given by WL, JW and GLH. Data acquisition was done by WL and JW. Quality control of data and algorithms was done by BL and JL. Data analysis, interpretation and statistical analysis were done by WL and JW.; Manuscript preparation was done by WL, MO and JD. Manuscript editing was done by JW, LS, CH and GLH. Manuscript review was done by GLH.

## Data availability

All data can be drawn from the dataset of the surveillance, epidemiology and end results (SEER) database (http://www.seer.cancer.gov).
