# Supplementary material for: Survival analysis between different treatment strategies of mixed adenoneuroendocrine carcinoma (MANEC): a population-based study
Source: Endocr Connect. 2025 Feb 18;14(3):e240350. doi: 10.1530/EC-24-0350 (PMC11850044; doi:10.1530/EC-24-0350)
Supplement: Supplementary file 1 [file supplementary_materials.pdf]

Supplementary Information

Table S1. Baseline clinicopathological characteristics of all patients

Figure S1. Plot of propensity score distribution before and after PSM for surgery

Figure S2. Plot of propensity score distribution before and after PSM for chemotherapy

Figure S3. Plot of propensity score distribution before and after PSM for radiation therapy

Table S1 Baseline clinicopathological characteristics of all patients (n=935)

Characteristics	cases (%)
Sex	
Male	481(51.4)
Female	454(48.6)
Age (years)	
< 60	447(47.8)
⩾ 60	488(52.1)
Race	
White	758(81.4)
Non-white	173(18.6)
Site	
Stomach	55(5.9)
Small Intestine	34(3.6)
Respiratory system	39(4.2)
Hepatic-biliary-pancreatic system	57(6.1)
Colorectum	223(23.9)
Appendix	482(51.6)
Other	45(4.8)
AJCC staging (6th)	
I+II	163(41.8)
III+IV	227(58.2)
Differentiation grade	
Grade I+II	149(30.8)
Grade III+IV	334(69.2)
Summary stage	
Localized	156(23.8)
Regional	258(39.4)
Distant	241(36.8)
Surgery	
No	120(12.8)
Yes	815(87.2)
Chemotherapy	
No/Unknown	440(47.1)
Yes	495(52.9)
Radiation therapy	
No/Unknown	872(93.5)
Yes	61(6.5)

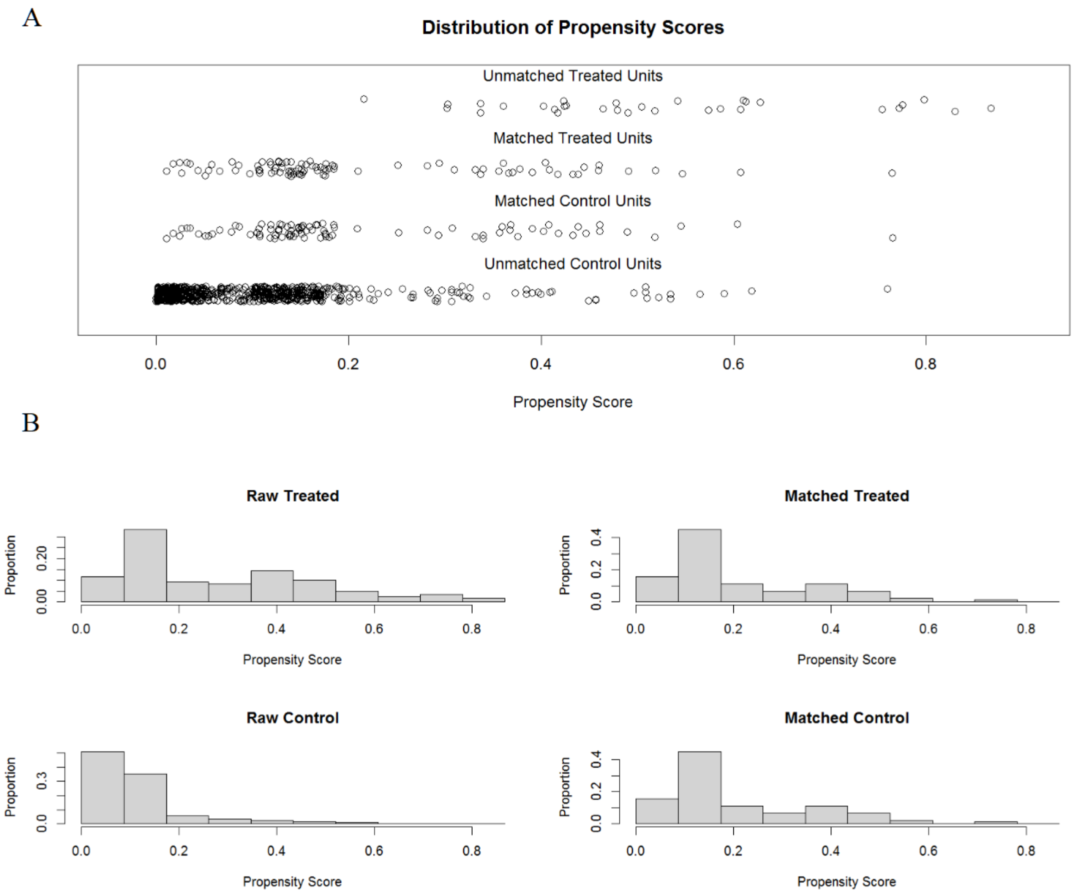

Figure S1. Plot of propensity score distribution before and after PSM for surgery.

A

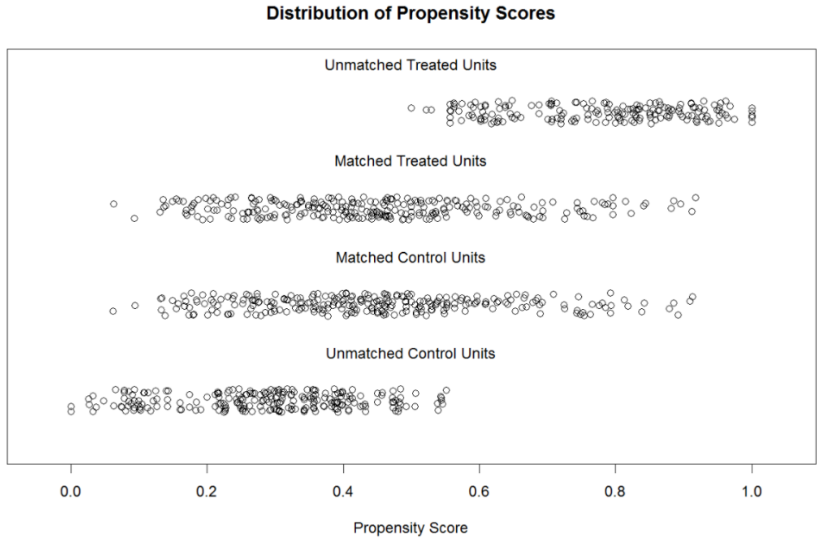

B

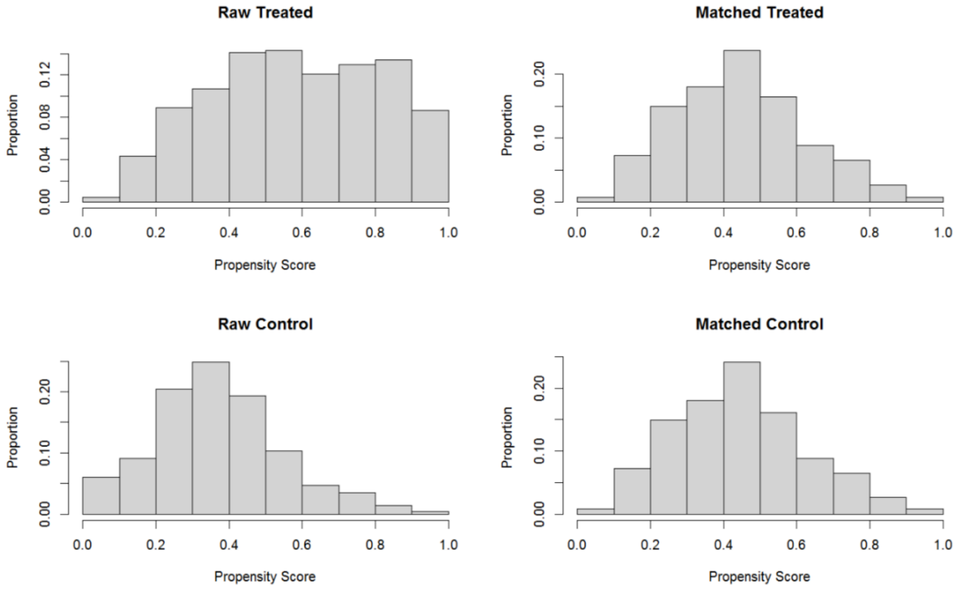

Figure S2. Plot of propensity score distribution before and after PSM for chemotherapy.

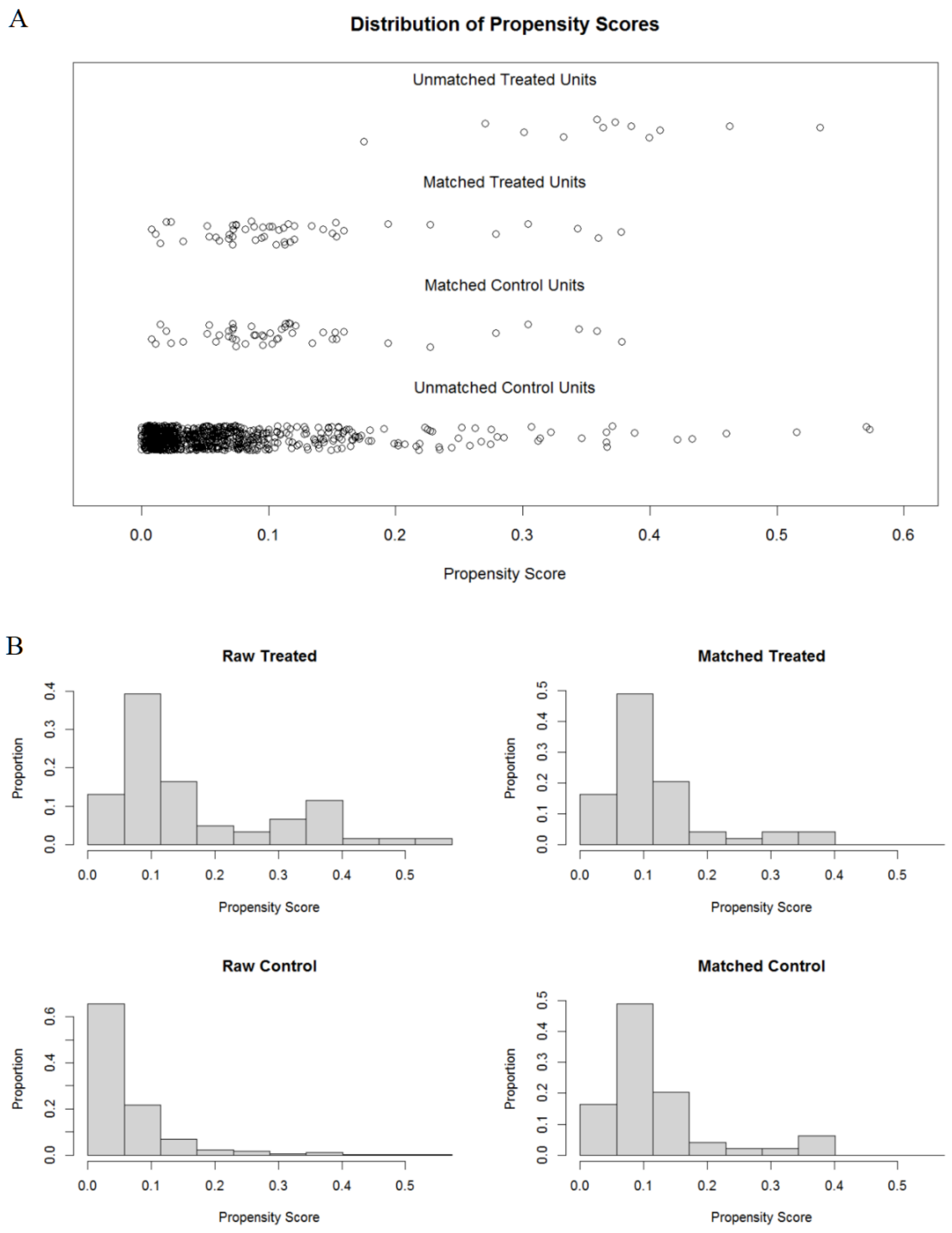

Figure S3. Plot of propensity score distribution before and after PSM for radiation therapy.
